# An oxidative stress biomarkers predict prognosis in gastric cancer patients receiving immune checkpoint inhibitor

**DOI:** 10.3389/fonc.2023.1173266

**Published:** 2023-07-20

**Authors:** Guiming Deng, Hao Sun, Rong Huang, Hongming Pan, Yanjiao Zuo, Ruihu Zhao, Zhongze Du, Yingwei Xue, Hongjiang Song

**Affiliations:** Department of Gastrointestinal Surgery, Harbin Medical University Cancer Hospital, Harbin Medical University, Harbin, Heilongjiang, China

**Keywords:** GIOSS, gastric cancer, ICIS, prognosis, PD-1/PD-L1

## Abstract

**Objective:**

The development and advance of gastric cancer are inextricably linked to oxidative and antioxidant imbalance. Although immunotherapy has been shown to be clinically effective, the link between oxidative stress and gastric cancer patients treated with immune checkpoint inhibitor (ICIs) remains unknown. This study aims at looking into the prognostic value of oxidative stress scores in gastric cancer patients treated with ICIs.

**Methods:**

By taking the propagation to receiver operating characteristic (ROC) we got the best cut-off values, and divided 265 patients receiving ICIs and chemotherapy into high and low GC-Integrated Oxidative Stress Score (GIOSS) groups. We also used Kaplan-Meier and COX regression models to investigate the relationship between oxidative stress biomarkers and prognosis.

**Results:**

Through both univariate and multivariate analyses, it’s shown that GIOSS severs as an independent prognostic factor for progression-free survival (PFS) and Overall survival (OS). Based on GIOSS cutoff values, patients with high GIOSS levels, compared to those with low levels exhibited shorter PFS and OS, both in the high GIOSS group, which performed poorly in the ICIs subgroup and other subgroup analyses.

**Conclusion:**

GIOSS is a biomarker that responds to systemic oxidative stress in the body and can predict prognosis in patients with gastric cancer who are taking ICIs. Additionally, it might come to medical professionals’ aid in making more effective or more suitable treatment plans for gastric cancer.

## Introduction

The importance of Helicobacter pylori screening as a significant risk factor for gastric cancer has been acknowledged, and the relative frequency and fatality of gastric cancer have decreased since Helicobacter pylori were eradicated (1, 2), but gastric cancer is still listed as the fifth most-seen cancer in the world (3). As scientific evidence builds up, multiple treatment modalities, such as surgery, chemotherapy, targeted therapy, and immunotherapy have been applied to patients with gastric cancer in later stages (2, 4–6). Meanwhile, a new era for immunotherapy has come, which contributes significantly to better the prognosis of advanced gastric cancer, for example, according to the clinical experiment of checkmate-649, Nivolumab shows superior OS, PFS benefit and an acceptable safety profile as the first PD-1 inhibitor. But in advanced gastric cancer, low five years survival rate remains a major challenge (7). Although, compared to that in urban areas, the mortality rate of gastric cancer has a better decline in rural areas, they are still the major region where gastric cancer is reported, which is closely related to their dietary patterns and environmental factors (8, 9). Therefore, it is necessary to find more convenient indicators to measure long-term prognosis in GC patients receiving immune checkpoint inhibitor (ICIs).

Based on precedent research, the entire process of carcinogenesis, progression, and death depends heavily on reactive oxygen species (ROS) (10, 11). However, gastric cancer has a markedly raised ROS level, which leads to genotoxicity and generates DNA damage. Meanwhile, Helicobacter pylori, a risk factor for gastric cancer, aids in the development of the disease by activating a number of oxidant-producing enzymes (12–14). Furthermore, the living environment and personal habits have a direct impact on the likelihood of gastric cancer, for example, increased levels of ROS and a high risk of gastric cancer can result from smoking, drinking alcohol, and being around biomass fumes (15–17).

In a study using a sleep-deprived mouse model to test oxidative stress and inflammation status, total bilirubin (TBIL), lactate dehydrogenase (LDH), blood urea nitrogen (BUN), and creatinine (CRE) levels were discovered to be significantly increased (18). Yinghao, Cao et al, created an oxidative stress index-based score for clinical outcomes in Colorectal Cancer, The CRC-Integrated Oxidative Stress Score (CIOSS) was composed of albumin (ALB), direct bilirubin (DBIL), and blood urea nitrogen (BUN) (19). All of these findings imply that biochemical indicators may serve as biomarkers for systemic oxidative stress. Oxidative stress was very intimately associated with the therapy of stomach cancer. For instance, in a study to investigate the impact of oxidative stress on gastric cancer, Luca Savino et al. found that the combination of H2O2 therapy with P13kk/k/ATK and MEK inhibitors reduced the survival of cancer cells (20). A ketogenic diet with antioxidant characteristics is also an effective course of treatment for stomach cancer, according to the research of Qiuju Xiao et al. (21). In light of this, we proposed that the GC-Integrated Oxidative Stress Score (GIOSS) could indicate the degree of inflammation and oxidative stress and determine whether gastric cancer patients with ICIs will benefit.

GIOSS is the most eminent evaluating technique to find out how cancer patients’ morbidity and mortality are contributed to oxidative stress status. In this study, we significantly analyzed the predictive ability of GIOSS for the survival expectancy of patients with ICIs by having 265 gastric cancer patients participate in the study at our institution. Meanwhile, by conducting subgroup analysis and building nomograms, we also tried to test and verify the ability of GIOSS to effectively predict the prognosis of gastric cancer.

## Materials and methods

### Patients

A sum of 265 people who received chemotherapy and ICIs between September 2016 to December 2022 were included in the retrospective study, 174 of whom received PD-1 inhibitor medication. All patients and their clinical data are assessed in compliance with the Declaration of Helsinki and its revisions. Detailed clinical information recorded from the patient’s electronic medical record includes age, gender, tumor site, TNM typing, tumor grading, and blood biochemical indexes. Patients with missing data or abnormal data were not included in the study.

Participants who meet the following criteria are included in: (1) pathological analysis supported the gastric cancer diagnosis. (2) there were no chronic diseases and cancers in any of the patients. (3) ICIs or chemotherapy were given to all participants. Participants without complete clinical information, not reviewed regularly after treatment and abandoned treatment will be excluded. Informed consent was given up by the Ethics Committee of Harbin Medical University Cancer Hospital because of the retrospective nature of this study.

### Date collection

Patients’ PFS and OS were obtained *via* telephone follow-up after clinical data collection. This study was last followed up on November 5, 2022. PFS was defined as the period of time starting from the beginning of the procedure, ICIs, or chemotherapy to the time when the disease started to advance. A computed tomography scan or chest and abdomen X-rays were used to detect progression. Patients without signs of progression at the time of death or at the final follow-up visit were included in the PFS definition. The OS was comprehended as the period of time between the first day of ICIs, surgery, or chemotherapy and the patient’s passing or last follow-up. The cut-off point is obtained for GIOSS by ROC, and they are then split into high and low value groups in accordance with cut-off point.

### Statistical analysis

Discrepancies between the two group were compared by Chi-square or Fisher’s exact test, survival was computed using Kaplan-Meier survival curves and differences in survival time between the two groups were assessed using Log-rank test. For oxidative stress-related biochemical indicators, they were first normalized and then subjected to ROC analysis. The hazard ratio (HR) and 95% confidence interval were used to evaluate relative risk (CI). It was decided to investigate the independent prognostic factors using a Cox proportional hazards regression model. Finally, a nomograms based on independent prognostic criteria was created to forecast the likelihood that PFS and OS will survive. R 4.1.3 (Vienna, Austria), and SPSS 25.0 (Chicago, IL, USA) were used for all statistical analysis (Vienna, Austria). Statistical differences were judged to exist when two-sided P values were 0.05.

## Results

### GIOSS established

The GIOSS systemic oxidative stress grading is made up of Oxidative stress-related parameters. We dichotomized the oxidative stress indicators according to the ROC cutoff values in order to investigate the prognostic significance of the oxidative stress grading. Univariate and multivariate Cox proportional-hazards regression model analysis of biochemical indicators that have been shown to be associated with oxidative stress in previous studies. Factors with P-values lower than 0.2 were put undergone multivariate analysis, and we found that the P-values of ALB, CRE, and IDBIL were lower than 0.2. These three’s regression coefficients were used for the needs of creating equations. GIOSS = 0.528 × IDBIL - 0.626 × CRE - 0.747 × ALB was the final result (**Table 1**). To simplify the calculation, we defined the oxidative stress indices as 1 if it’s more than the cut-off value and 0 if it’s less than the cut-off value.

**Table 1:** Multivariate Cox regression analysis determined GIOSS composition indicators.

Parameters	β	OS	P value	β	OS	P value
		Univariate analysis			Multivariate analysi	
		Hazard ratio (95%CI)			Hazard ratio (95%CI)	
ALB	-0.703	0.495 (0.330-0.742)	0.001	-0.747	0.474 (0.312-0.720)	<0.001
CRE	-0.744	0.475 (0.307-0.734)	0.001	-0.626	0.535 (0.314-0.840)	0.007
IDBIL	0.329	1.389 (0.935-2.064)	0.104	0.538	1.712 (1.141-2.570)	0.009
Urea	-0.521	0.594 (0.352-1.002)	0.051	-0.314	0.730 (0.427-1.248)	0.250
DBIL	0.237	1.267 (0.764-2.101)	0.358			

### Patient characteristics

The median age of the 265 gastric cancer patients, at age of ranging from 32 to 82, in this retrospective cohort analysis was 59 years. Among them, 182 (68.7%) were males, and 83 (21.3%) were females. In this study, 117 patients received adjuvant therapy, while the rest 148 patients (55.8%) underwent surgical treatment. Additionally, 174 (65.7%) individuals received ICIs treatment. 148 (55.8%) patients were included in the low GIOSS group and 117 (44.2%) patients were included in the high GIOSS group based on the optimal cut-off value of -0.69, which was established by ROC. The chi-square test results revealed a relationship between GIOSS and BMI (p = 0.047), surgery (p = 0.005), pathology (p = 0.010), and TNM stage (p = 0.006). The specific clinical attributes of all 265 cases classified by GIOSS are demonstrated in **Table 2**.

**Table 2:** The clinical information of all patients.

N	level	Low GISOO	High GISOO	p
		148	117	
Sex(%)	Male	106 (71.6)	76 (65.0)	0.245
	Female	42 (28.4)	41 (35.0)	
Age (%)	<59	79 (53.4)	53 (45.3)	0.191
	≥59	69 (46.6)	64 (54.7)	
BMI (%)	<21.69 (kg/m_2_)	66 (44.6)	66 (56.9)	0.047
	≥21.69 (kg/m_2_)	82 (55.4)	50 (43.1)	
SLNM (%)	No	126 (85.1)	93 (79.5)	0.228
	Yes	22 (14.9)	24 (20.5)	
ECG (%)	Normal	35 (23.6)	20 (17.1)	0.191
	Abnormal	113 (76.4)	97 (82.9)	
Surgery (%)	Yes	94 (63.5)	54 (46.2)	0.005
	No	54 (36.5)	63 (53.8)	
Primary tumor site (%)	Upper 1/3	84 (56.8)	57 (48.7)	0.623
	Middle 1/3	52 (35.1)	46 (39.3)	
	Low 1/3	11 (7.4)	13 (11.1)	
	Whole	1 (0.7)	1 (0.9)	
Borrmann type (%)	Borrmann I + II	6 (4.1)	4 (3.4)	0.886
	Borrmann III + IV	22 (14.8)	15 (12.8)	
	Unknown	120 (81.1)	98 (83.8)	
Tumor size (%)	<50 mm	34 (23.0)	16 (13.7)	0.147
	≥50 mm	16 (10.8)	16 (13.7)	
	Unknown	98 (66.2)	85 (72.6)	
Pathology (%)	Adenocarcinoma	59 (39.9)	34 (29.1)	0.010
	Others	16 (10.8)	5 (4.3)	
	Unknown	73 (49.3)	78 (66.6)	
TNM stage (%)	I + II	27 (18.2)	28 (6.8)	0.006
	III + IV	121 (81.8)	109 (93.2)	
Differentiation (%)	Poor	74 (38.3)	21 (29.2)	0.166
	Moderately + Well	119 (61.7)	51 (70.8)	
Lauren type (%)	Intestinal	12 (8.1)	6 (5.1)	0.218
	Diffuse	13 (8.8)	5 (4.3)	
	Mixed	16 (10.8)	9 (7.7)	
	Unknown	107 (72.3)	97 (82.9)	
PD-1 (%)	Positive	24 (16.2)	18 (15.4)	0.514
	Negative	57 (38.5)	38 (32.5)	
	Unknown	67 (45.3)	61 (52.1)	
PD-L1 (%)	Positive	40 (28.4)	25 (26.5)	0.425
	Negative	42 (27.0)	31 (21.4)	
	Unknown	66 (44.6)	61 (52.1)	
Treatment (%)	ICIs	90 (60.8)	84 (71.8)	0.062
	Chemotherapy	58 (39.2)	33 (28.2)	

#BMI, body mass index; ECG, electrocardiogram; SLNM, supraclavicular lymph node; Others of Pathology, include mucinous carcinoma, signet ring cell carcinoma, mixed carcinoma, unknown.

### Blood parameters

We utilized the chi-square test and Fisher’s exact test to examine the relationship between patients’ pre-treatment blood test results and GIOSS by dividing them into two groups based on the median. The results demonstrated that, from all enrolled patients, alanine aminotransferase (ALT) (p = 0.019), aspartate aminotransferase (AST) (p = 0.020), alkaline phosphatase (ALP) (p = 0.043), TP (p < 0.001), prealbumin (PALB) (p < 0.001), monocytes (Mono) (p = 0.007), basocytes (Baso) (p=0.007), red blood cell (RBC) (p < 0.001), hemoglobin (Hb) (p < 0.001) and carbohydrate antigen 724 (CA724) (p = 0.024) were statistically significant differences between the two GIOSS groups, while other parameters were statistically insignificant differences between the two GIOSS groups (P > 0.05) in **Table 3**.

**Table 3:** The blood parameters of all patients.

N	level	Low GIOSS	High GIOSS	p
		148	117	
ALT (%)	<14 U/L	62 (41.9)	66 (56.4)	0.019
	≥14 U/L	86 (58.1)	51 (43.6)	
AST (%)	< 20 U/L	61 (41.2)	65 (55.6)	0.020
	≥ 20 U/L	87 (58.8)	52 (44.4)	
γ-GGT (%)	< 23 U/L	72 (48.6)	57 (48.7)	0.991
	≥ 23 U/L	76 (51.4)	70 (51.3)	
LDH (%)	<174 U/L	75 (50.7)	57 (48.7)	0.752
	≥174 U/L	73 (49.3)	60 (51.3)	
ALP (%)	<90 U/L	65 (43.9)	66 (56.4)	0.043
	≥90 U/L	83 (56.1)	51 (43.6)	
TBIL (%)	<11.5 umol/L	67 (45.3)	65 (55.6)	0.096
	≥11.5 umol/L	81 (54.7)	52 (44.4)	
IDBIL (%)	<9.0 umol/L	68 (45.9)	64 (54.7)	0.157
	≥9.0 umol/L	80 (54.1)	53 (45.3)	
TP (%)	<69.1 g/L	45 (30.4)	86 (73.5)	<0.001
	≥69.1 g/L	103 (69.6)	31 (26.5)	
PALB (%)	<204 g/L	49 (33.1)	82 (70.1)	<0.001
	≥204 g/L	99 (66.9)	35 (29.9)	
GLOB (%)	<30.0 g/L	73 (49.3)	59 (50.4)	0.858
	≥30.0 g/L	75 (50.7)	58 (49.6)	
WBC (%)	<6.37 109/L	72 (48.6)	58 (49.6)	0.881
	≥6.37 109/L	76 (51,4)	59 (50.4)	
Neu (%)	<3.97 109/L	75 (50.7)	57 (48.7)	0.752
	≥3.97 109/L	73 (49.3)	60 (51.3)	
Mono (%)	<0.49 109/L	84 (56.8)	47 (40.2)	0.007
	≥0.49 109/L	64 (43.2)	70 (59.8)	
Eosi (%)	<0.1 109/L	68 (45.9)	57 (48.7)	0.654
	≥0.1 109/L	80 (54.1)	60 (51.3)	
Baso (%)	<0.02 109/L	30 (20.3)	35 (29.9)	0.070
	≥0.02 109/L	118 (79.7)	82 (70.1)	
RBC (%)	<4.33 1012/L	51 (34.5)	79 (67.5)	<0.001
	≥4.33 1012/L	97 (65.5)	38 (32.5)	
L (%)	<1.6 109/L	70 (47.3)	62 (53.0)	0.357
	≥1.6 109/L	78 (52.7)	58 (47.0)	
Hb (%)	<124.2 g/L	80 (41.5)	52 (47.0)	<0.001
	≥124.2 g/L	113 (58.5)	20 (27.8)	
P (%)	<243 109/L	79 (53.4)	53 (45.3)	0.191
	≥243 109/L	69 (46.6)	64 (54.7)	
CEA (%)	<3.18 ng/mL	81 (54.7)	51 (43.6)	0.072
	≥3.18 ng/mL	67 (45.3)	66 (56.4)	
AFP (%)	<2.80 ng/mL	70 (47.3)	62 (53.0)	0.257
	≥2.80 ng/mL	78 (52.7)	55 (47.0)	
CA199 (%)	<16.14 U/mL	77 (52.0)	55 (47.0)	0.417
	≥16.14 U/mL	71 (48.0)	62 (53.0)	
CA724 (%)	<4.16 U/mL	82 (55.4)	48 (41.4)	0.024
	≥4.16 U/mL	66 (44.6)	68 (58.6)	
CA125II (%)	<23.90 U/mL	73 (49.3)	58 (49.6)	0.968
	≥23.90 U/mL	75 (50.7)	59 (50.4)	

### Univariate and multivariate cox regression survival analyses for survival analysis

We studied the impact of baseline features and GIOSS on the clinical outcomes of gastric cancer by using univariate and multivariate COX proportional-hazards models and then discover independent prognostic factors. As is shown in **Table 4**, as univariate analysis concluded, the prognosis factors of patients in this study for PFS were ALP (HR = 1.532, p = 0.037), indirect bilirubin (IDBIL) (HR = 1.644, p = 0.016), Total protein (TP) (HR = 0.650, p = 0.035), PALB (HR = 0.594, p = 0.011), carcinoembryonic antigen (CEA) (HR = 1.645, p = 0.017), CA724 (HR = 1.954, p = 0.002), TNM stage (HR = 2.754, p = 0.010) and GIOSS (HR = 2.363, p < 0.001). The prognosis factors of patients in this study for OS were TP (HR = 0.668, p = 0.048), PALB (HR = 0.590, p = 0.010), CEA (HR = 1.831, p = 0.004), CA724 (HR = 2.097, p < 0.001), TNM stage (HR = 3.251, p = 0.003) and GIOSS (HR = 2.554, p < 0.001). The multivariate analysis revealed that ALP (HR = 1.674, p = 0.013), CA724 (HR = 1.551, p = 0.046), and GIOSS (HR = 1.848, p = 0.016) were independent prognostic factors for PFS. The multivariate analysis indicated CA724 (HR = 1.598, p = 0.032), TNM stage (HR = 2.448, p = 0.027), and GIOSS (HR = 2.016, p = 0.005) as independent prognostic factors for OS. Moreover, we found that CA724 is an independent prognostic factor both for PFS and OS.

**Table 4:** Univariate and multivariate analysis for PFS and OS.

Parameters	Univariate analysis	PFS	Multivariate analysi	P value	Univariate analysis	OS	Multivariate analysi	P value
		P value				P value		
	Hazard ratio (95%CI)		Hazard ratio (95%CI)		Hazard ratio (95%CI)		Hazard ratio (95%CI)	
Sex (Male vs Female)	0.924 (0.602-1.419)	0.717			0.989 (0.644-1.518)	0.959		
Age (<59 vs ≥59)	1.011 (0.681-1.501)	0.958			0.949 (0.639-1.409)	0.794		
ALT (<14 U/L vs ≥14 U/L)	0.941 (0.632-1.399)	0.763			0.901 (0.605-1.343)	0.609		
AST (<20 U/L vs ≥20 U/L)	1.168 (0.785-1.738)	0.444			1.238 (0.832-1.842)	0.292		
CGT (<23U/L vs ≥23 U/L)	1.061 (0.715-1.574)	0.771			1.117 (0.753-1.658)	0.582		
LDH (<174 U/L vs ≥174 U/L)	1.074 (0.723-1.596)	0.723			1.276 (0.857-1.901)	0.230		
ALP (<90 U/L vs ≥90 U/L)	1.532 (1.027-2.286)	0.037	1.674 (1.113-2.520)	0.013	1.390 (0.931-2.075)	0.107		
IDBIL (<9.00 umol/L vs ≥9.00 umol/L)	1.644 (1.097-2.463)	0.016			1.481 (0.990-2.214)	0.056		
TP (<69.1 g/L vs ≥69.1 g/L)	0.650 (0.436-0.969)	0.035	0.852 (0.540-1.344)	0.491	0.668 (0.448-0.996)	0.048	1.004 (0.630-1.601)	0.987
GLOB (<30.0 g/L vs ≥30.0 g/L)	0.905 (0.610-1.344)	0.622			1.015 (0.683-1.507)	0.942		
PALB (<204 mg/Lvs ≥204 mg/L)	0.594 (0.397-0.888)	0.011	0.827 (0.530-1.289)	0.402	0.590 (0.394-0.883)	0.010	0.792 (0.512-1.223)	0.293
HB (<124.2 g/L vs ≥124.2 g/L)	0.918 (0.619-1.362)	0.670			0.929 (0.626-1.378)	0.713		
N (<3.97 10^9^/L vs ≥3.97 10^9^/L)	1.300 (0.875-1.931)	0.195			1.455 (0.997-2.166)	0.065		
L (<1.6 10^9^/L vs ≥1.6 10^9^/L)	0.751 (0.502-1.122)	0.162			0.693 (0.465-1.031)	0.071		
M (<0.49 10^9^/L vs ≥0.49 10^9^/L)	1.166 (0.782-1.740)	0.452			1.088 (0.731-1.619)	0.679		
AFP (<2.8 ng/mL vs ≥2.8 ng/mL)	0.757 (0.509-1.127)	0.170			0.775 (0.521-1.153)	0.209		
CEA (<3.18 ng/mL vs ≥3.18 ng/mL)	1.645 (1.093-2.478)	0.017	1.375 (0.897-2.107)	0.144	1.831 (1.219-2.752)	0.004	1.442 (0.937-2.220)	0.096
CA199 (<16.14 U/mL vs ≥16.14 U/mL)	1.257 (0.845-1.869)	0.258			1.457 (0.978-2.170)	0.064		
CA724 (<4.16 U/mL vs ≥4.16 U/mL)	1.954 (1.287-2.967)	0.002	1.551 (1.007-2.338)	0.046	2.097 (1.392-3.160)	<0.001	1.598 (1.042-2.452)	0.032
CA125II (<23.90 U/mL vs ≥23.90 U/mL)	1.282 (0.862-1.906)	0.220			1.115 (0.752-1.654)	0.589		
Borrmann type (I + II vs III + IV + Unknown)	1.191 (0.377-3.761)	0.776			1.040 (0.329-3.286)	0.946		
TNM stage (I + II vs III + IV)	2.754 (1.268-5.978)	0.010	2.016 (0.909-4.472)	0.085	3.251 (1.494-7.074)	0.003	2.448 (1.106-5.419)	0.027
GIOSS (<-0.69 vs ≥-0.69)	2.363 (1.566-3.567)	<0.001	1.848 (1.122-3.043)	0.016	2.554 (1.699-3.839)	<0.001	2.016 (1.236-3.291)	0.005

### Survival for GIOSS

**Figure 1:**
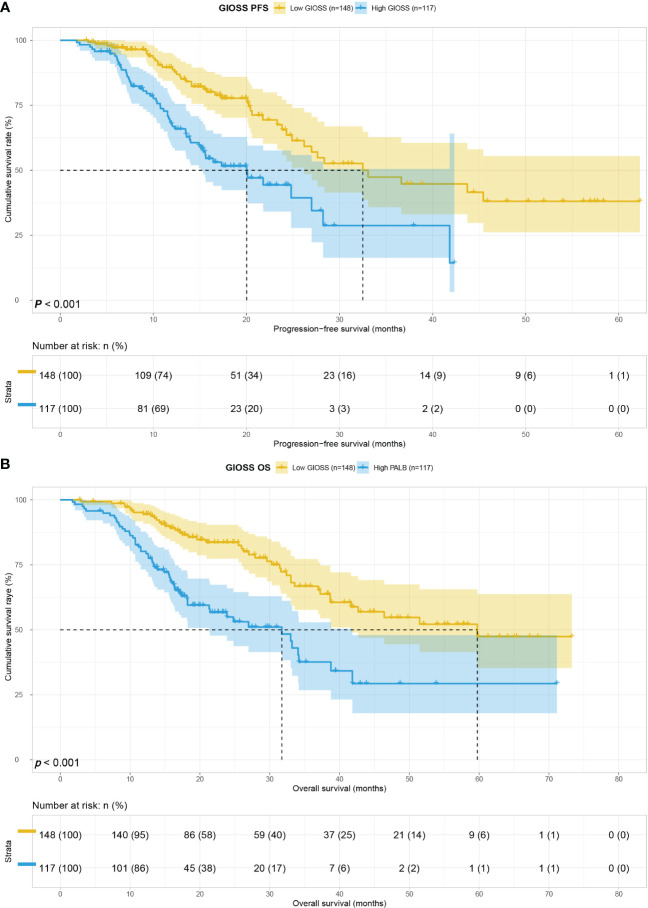
GIOSS related survival curve of PFS **(A)** and OS **(B)** in all patients.

Patients in high GIOSS group had median PFS and OS time of 20.03 and 31.73 months, while patients in low GIOSS group had median PFS and OS time of 32.50 and 59.73 months. Furthermore, the log-rank analysis concluded that the median PFS and OS survival time of GIOSS in high group were noticeably shorter than those of GIOSS in low group (HR = 2.363, p < 0.001, and HR = 2.545, p < 0.001). **(** **Figures 1A, B** **).**

### Survival for treatment

**Figure 2:**
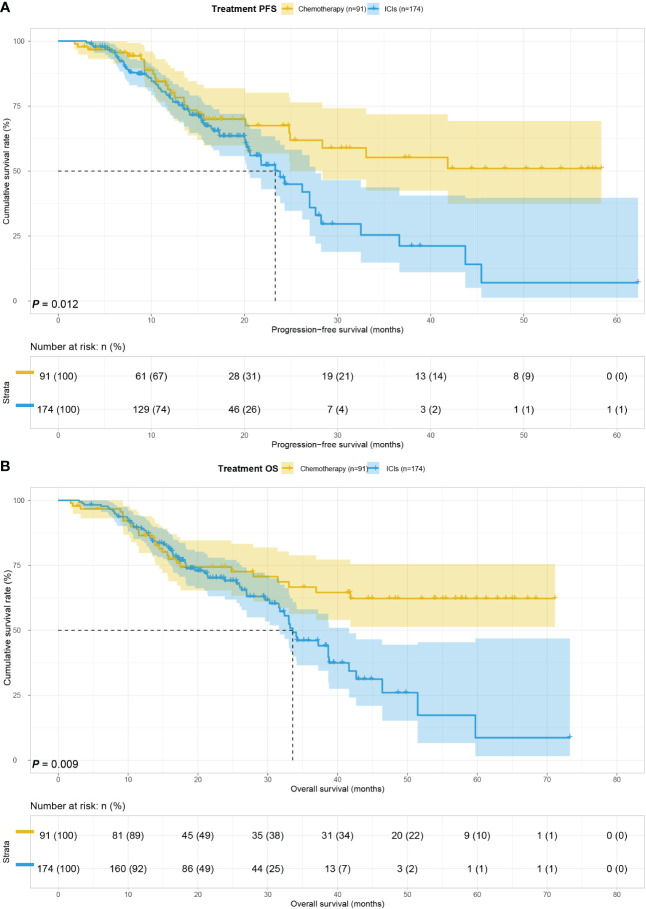
Treatment related survival curve of PFS **(A)** and OS **(B)** in all patients.

In order to further validate the prognostic role of GIOSS on patients with ICIs of gastric cancer, we split the 265 patients into ICIs group (174 patients) and chemotherapy group (91 patients). The association between baseline features and treatment was then studied. Between two different treatment groups, we discovered that Surgery (p < 0.001), Tumor size (p = 0.001), Pathology (p < 0.001), TNM stage (p < 0.001), Differentiation (p < 0.001), Lauren type (p < 0.001), PD-1 (p < 0.001), PD-L1 (p < 0.001) had statistically significant differences **(** **Table 5** **)**. The median survival time (MST) for PFS and OS in ICIs group were 23.31 and 33.57 months, and the MST for PFS and OS in chemotherapy group were both not reached. Both PFS (HR = 1.805, p = 0.012) and OS (HR = 1.841, P = 0.009) were significantly better in patient receiving chemotherapy than in patients with ICIs. **(** **Figures 2A, B** **)**.

**Table 5:** The clinical information for treatment.

n	level	Chemotherapy	ICIs	p
		91	174	
Sex (%)	Male	58 (63.7)	124 (71.3)	0.210
	Female	33 (36.3)	50 (28.7)	
Age (%)	<59	45 (49.5)	87 (50.0)	0.932
	≥59	46 (50.5)	87 (50.0)	
BMI (%)	<21.69 (kg/m_2_)	48 (53.3)	84 (48.3)	0.436
	≥21.69 (kg/m_2_)	42 (46.7)	90 (51.7)	
SLNM (%)	No	78 (85.7)	141 (81.0)	0.340
	Yes	13 (14.3)	33 (19.0)	
ECG (%)	Normal	19 (20.9)	36 (20.7)	0.971
	Abnormal	72 (79.1)	138 (79.3)	
Surgery (%)	Yes	72 (79.1)	76 (43.7)	<0.001
	No	19 (20.9)	98 (56.3)	
Primary tumor site (%)	Upper 1/3	53 (58.2)	88 (50.6)	0.076
	Middle 1/3	25 (27.5)	73 (42.0)	
	Low 1/3	12 (13.2)	12 (6.8)	
	Whole	1 (1.1)	1 (0.6)	
Borrmann type (%)	Borrmann I + II	2 (2.2)	8 (4.6)	0.573
	Borrmann III + IV	14 (15.4)	23 (13.2)	
	Unknown	75 (82.4)	143 (82.2)	
Tumor size (%)	<50 mm	28 (30.8)	22 (12.6)	0.001
	≥50 mm	13 (14.3)	19 (10.9)	
	Unknown	50 (54.9)	133 (76.4)	
Pathology (%)	Adenocarcinoma	53 (58.2)	40 (23.0)	<0.001
	Others	10 (2.6)	11 (6.3)	
	Unknown	28 (30.8)	123 (70.7)	
TNM stage (%)	I + II	24 (26.4)	11 (6.3)	<0.001
	III + IV	67 (73.6)	163 (93.7)	
Differentiation (%)	Poor	53 (58.2)	42 (24.1)	<0.001
	Moderately + Well	38 (41.8)	132 (75.9)	
Lauren type (%)	Intestinal	12 (13.2)	6 (3.4)	<0.001
	Diffuse	14 (15.4)	4 (2.3)	
	Mixed	16 (17.6)	9 (5.2)	
	Unknown	49 (53.8)	155 (89.1)	
PD-1 (%)	Positive	30 (33.0)	12 (6.9)	<0.001
	Negative	59 (64.8)	36 (20.7)	
	Unknown	2 (2.2)	126 (72.4)	
PD-L1 (%)	Positive	31 (63.7)	34 (19.5)	<0.001
	Negative	58 (34.1)	15 (8.6)	
	Unknown	2 (2.2)	125 (71.8)	

**Figure 3:**
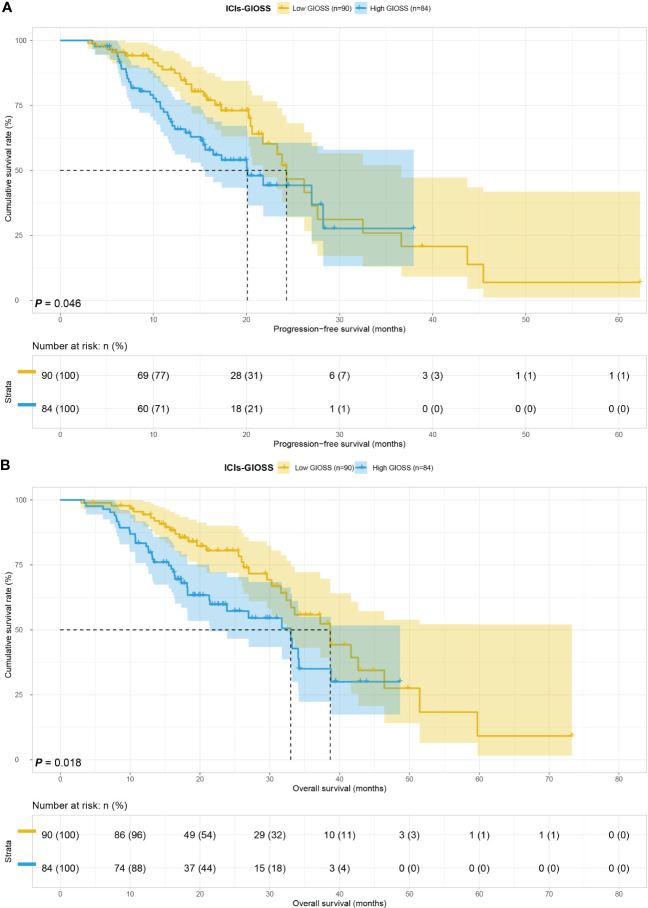
GIOSS related survival curve of PFS **(A)** and OS **(B)** in ICIs group.

The ICIs group included 84 people with the high level of GIOSS and 90 people with the low level of GIOSS. For the low GIOSS and high GIOSS groups, the MST for PFS was 24.30 and 20.11 months respectively, while the MST for OS in the two groups was 38.67 and 33.00 months. It is evident that the MST for PFS (p = 0.046) and OS (p = 0.018) were higher in the low GIOSS group than in the high GIOSS group. **(** **Figures 3A, B** **)**.

**Figure 4:**
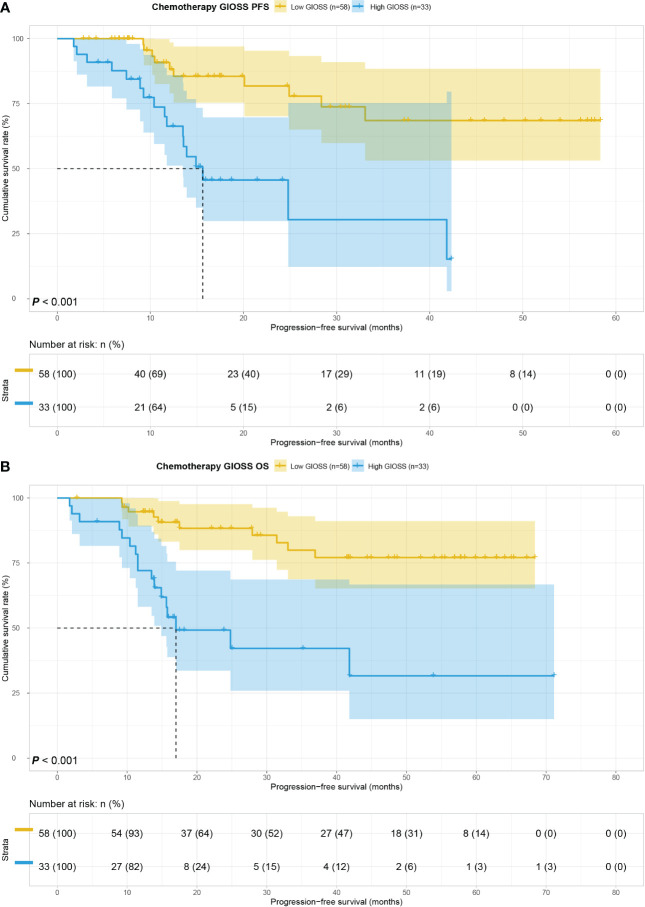
GIOSS related survival curve of PFS **(A)** and OS **(B)** in chemotherapy group.

Of all those who received chemotherapy, 58 cases were appointed to the low GIOSS group while 33 cases to the high GIOSS group. Univariate analyses: The same conclusion was drawn in the chemotherapy group, where patients in the low GIOSS group had superior OS (p < 0.001) and PFS (p < 0.001). The MST for PFS and OS in the high GIOSS group were 15.63 and 17.00 months, and neither PFS nor OS in low GIOSS group reached the MST. **(** **Figures 4A, B** **)**.

### Subgroup analysis of TNM stage

**Figure 5:**
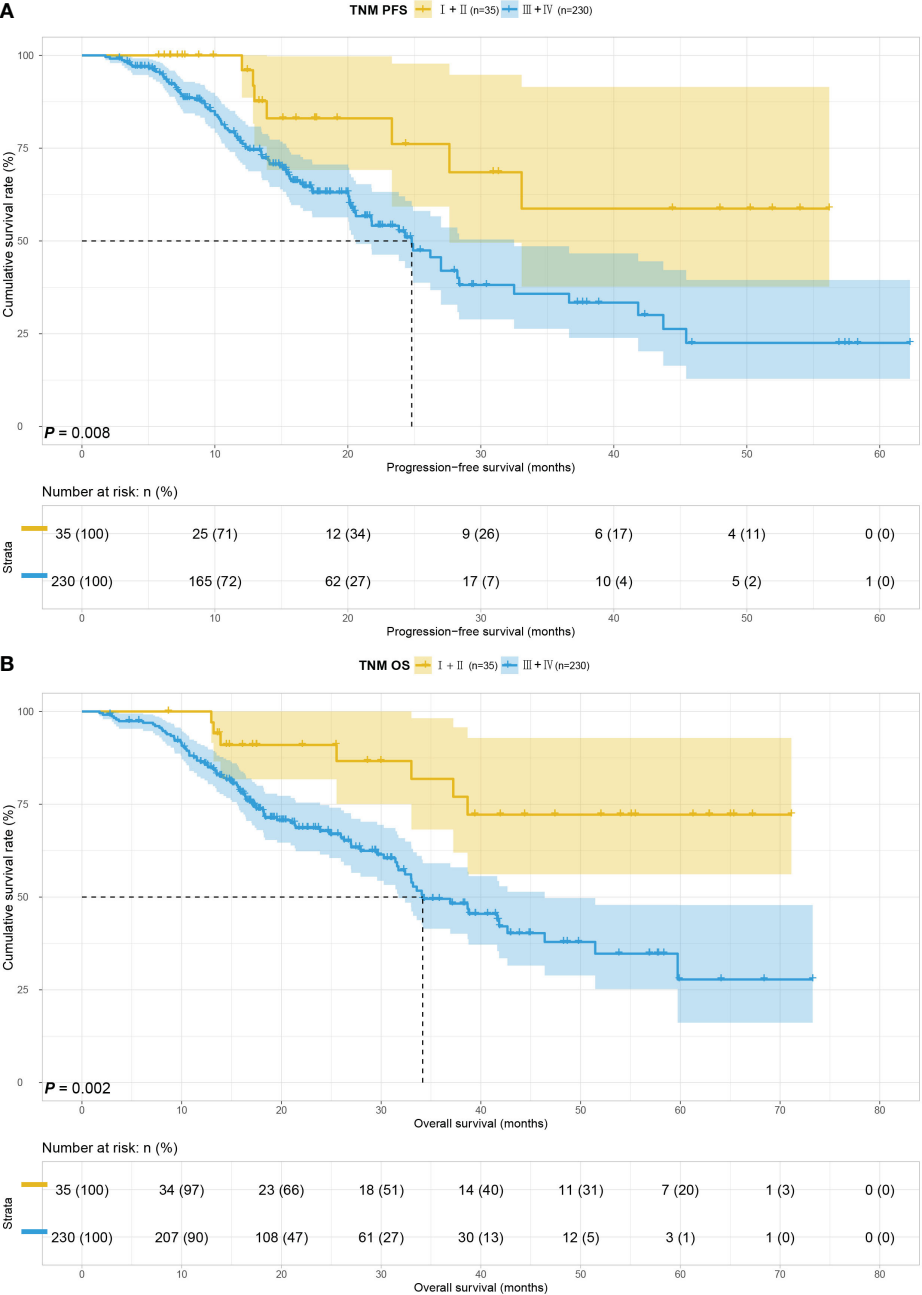
TNM stage related survival curve of PFS **(A)** and OS **(B)** in all patients.

**Figure 6:**
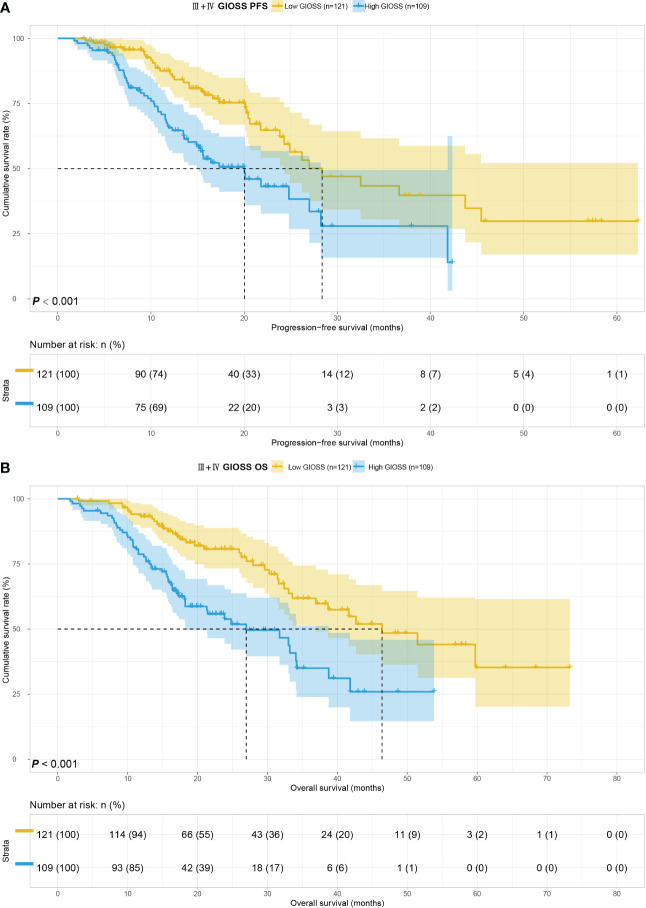
GIOSS related survival curve of PFS **(A)** and OS **(B)** in III+IV group.

**Figure 7:**
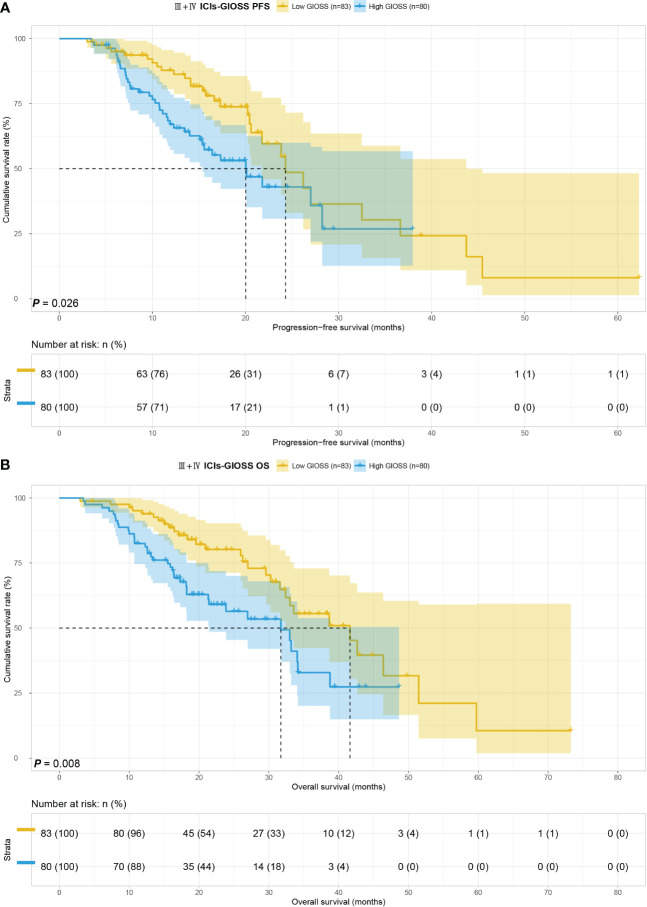
GIOSS related survival curve of PFS **(A)** and OS **(B)** in III+IV ICIs group.

We considered the TNM stage as an important prognostic factor for tumor patients, and subgroup analysis of the TNM stage was carried out. We performed a subgroup analysis on just III+IV stage patients in this study because III+IV stage patients are the majority of the study’s patients. In order to further look into the predictive value of GIOSS in immunotherapy, we performed a subgroup analysis of patients with ICIs in advanced stages. Kaplan-Meier analysis: the I + II stage group compared to the III+IV stage group showed longer PFS (p = 0.008) and OS (p = 0.002) **(** **Figures 5A, B** **)**. Subsequently, we explored the prognosis of GIOSS in the III+IV stage subgroup. Patients with high GIOSS showed shorter PFS (p < 0.001) and OS (p < 0.001) than patients with low GIOSS **(** **Figures 6A, B** **)**. In the subgroup analysis of patients with ICIs at advanced stages, the result showed that patients with low GIOSS had longer OS (p = 0.008) and PFS (p=0.026) survival time than those with high GIOSS. **(** **Figures 7A, B** **)**.

### Time-dependent ROC analysis

**Figure 8:**
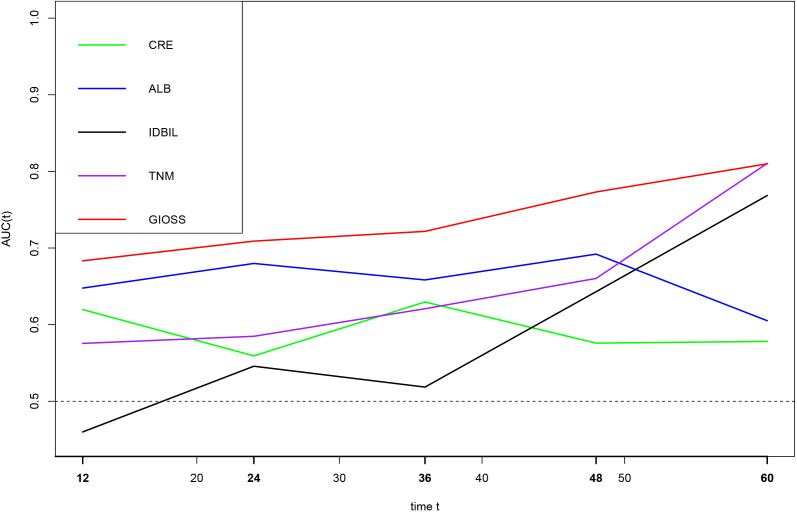
Time-dependent ROC analysis of CRE, ALB, IDBIL, GIOSS, and TNM stages on clinical outcomes in 265 GC patients.

We performed a time-dependent ROC to deeply evaluate the predictive significance of prognostic parameters linked to oxidative stress. The findings demonstrated that among the prognostic markers of, IDBIL, ALB, CRE, TNM stage and GIOSS, the AUC of GIOSS were higher than those of IDBIL, ALB, CRE and TNM stage in five years. Additionally, we found that the AUC of GIOSS was largely steady in five years. **(** **Figure 8** **)**.

### Assessment of the constructed nomograms

**Figure 9:**
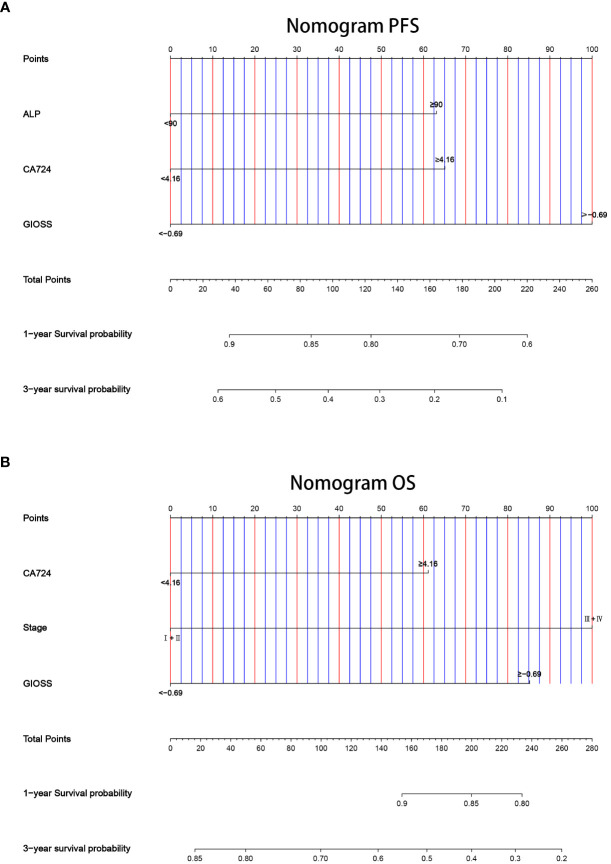
Nomogram for predicting 1- and 3-year survival probability of PFS **(A)** and OS **(B)**.

We chose PFS and OS independent prognostic parameters based on the outcomes of multivariate analysis to construct prognostic nomograms for gastric cancer and the precision of prediction was assessed by Harrell’ s c-index. The nomogram for PFS included ALP, CA724 and GIOSS, whereas the nomogram for OS included CA724, TNM stage and GIOSS. The 1-year and 3-year survival rates of PFS (c-index: 0.666) and OS (c-index: 0.682) were examined by the nomogram **(** **Figures 9A, B** **)**.

## Discussion

The subject of this study is the importance of systemic oxidative stress status on the prognosis of gastric cancer patients who receive ICIs, which serves the starter who developed a predictive model that incorporates GIOSS. Patients are benefiting from the numerous PD-1/PD-L1 inhibitors that have been authorized and commercialized for extensive clinical usage. Large-scale clinical trials for renal cell carcinoma, bladder cancer, and Hodgkin’s Lymphoma are still continuing with PD-1 inhibitors, which are mostly for melanoma and non-small cell lung cancer. Atezolizumab, durvalumab, and avelumab are PD-L1 inhibitors that have been approved for the treatment of urothelial carcinoma, and numerous other medications are still undergoing preliminary clinical trials (22–25). In this study, we investigated the prognostic role of GIOSS in patients treated with ICIs, with the aim of benefiting patients with negative PD-L1 expression.

There has been research managing to predict patients’ prognosis by evaluating their oxidative stress status. For example, in 2018 the oxidative stress index (OSI) was utilized by Xuefang Du et al. to assess the prognosis of 284 patients following radical resection for primary stage III gastric cancer, and they found that preoperative OSI was a reliable predictor of prognosis for patients with operable and advanced gastric cancer (26). In 2021, Kaiming Zhang et al. developed an oxidative stress score (SOS) and built a predictive model based on it to examine the clinical outcomes of 1583 patients with operable breast cancer (27). In the same year, Yinghao Cao et al. also used laboratory indicators related to oxidative stress to analyze the prognosis of 1422 patients with surgically resectable CRC, and the results stated that CIOSS has prognostic value for CRC patients (19).

Gastric cancer development and progression are related to oxidative and antioxidant imbalance (28). Specifically speaking, high risk factors for gastric cancer including Helicobacter pylori infection, diet and lifestyle, and obesity, are closely linked to oxidants and oxidative stress (29, 30). Microsatellite instability (MSI)is a predictive biomarker for immunotherapy since High - Microsatellite Instability (MSI - H) phenotype leads to elevated PD-L1 expression in gastric cancer (31, 32). We can learn from a study that oxidative phosphorylation and reactive oxygen pathways promoted apoptosis, making an excellent prognosis over to gastric cancer patients possessing the MSI phenotype (33). Oxaliplatin is functioning in the treatment of gastric cancer, and a good prognosis has been achieved in patients treated with the combination of oxaliplatin and PD-1 inhibitors, and it induces immunogenic death and stimulates an anti-tumor immune response (34, 35). However, the development of oxaliplatin resistance affected the subsequent treatment of patients, and The oxidative stress induced DNA damage repair response is found to be an important mechanism for inducing oxaliplatin resistance (36). From the perspective of high-risk factors of gastric cancer development and treatment, gastric cancer maintains a close bond to oxidative stress. Both excessive oxidants and factors which increase the degree of oxidative stress will add up to the chance of developing gastric cancer, and the oxidative stress status affects the effectiveness of oncologic drug therapy, leading to the promotion of tumor progression.

Inhibiting the generation of ROS can be crucial in the diagnosis and treatment of gastric cancer since gastric cancer tissues produce more ROS than healthy gastric tissues do (37). Inflammatory pathways are triggered by oxidative stress, which then encourages the development and spread of tumors (38). Among the indicators included in the study, the fundamental ideas of oxidative stress biomarkers and systemic inflammatory indicators are similar in some way. But they also play a crucial part in oxidative stress. In this study, we found that decreased ALB, an essential prognostic bio-inflammatory marker that responds to nutritional and inflammatory status as well as its antioxidant function, had connection to shorter OS in gastric cancer patients (39, 40). The antioxidant activity of albumin is due to its metal binding properties and the redox properties of Cys34 thiol (41). Nrf2 is an emerging antioxidant gene that can act as an intermediary and be activated by antioxidant compounds, thus contributing to the decline in CRE (42, 43). After generated from muscle metabolism, CRE was excreted through kidneys. Increased oxidative stress lowers the kidneys’ ability to act as antioxidants, which then impairs CRE excretion and raises blood CRE levels (44). In this study, CRE was a protective factor and gastric cancer patients with lower CRE were associated with longer OS. Unconjugated bilirubin (UCB), also in short as indirect bilirubin (IDBIL), is both an antioxidant and an indirect pro-oxidant. In Gianluca Tell’s article on UCB and oxidative stress, it is demonstrated that UCB induces the production of ROS, which makes the downstream APE1/Ref-1 convert to Egr-1 and further to PTEN (APE1/Ref-1 → Egr-1 →PTEN pathway) (45). Egr-1, a gene that suppress tumor growth, and PTEN, a new oncogene whose expression stops unchecked cell growth, thereby preventing the development of tumors (46, 47). In this study, we discovered a positive relevance between prognosis and high IDBIL.

This study primarily included biological indicators of oxidative stress. After conducting univariate and multivariate analyses, we chose to establish GIOSS grading with ALB, IDBIL, and CRE. We discovered that GIOSS severs as an independent prognostic factor in patients with gastric cancer in a multivariate analysis. For the first time, this study illustrated the tie linking systemic oxidative stress indicators to the prognosis of gastric cancer patients receiving immunotherapy.

There were few articles investigating the effect of oxidative stress status on the prognosis of tumor patients in the past two years. GIOSS is constructed by the same mechanism as SOS and CIOSS, both using common clinical laboratory indicators, and GIOSS systematically provides an oxidative stress status as a new biomarker of oxidative stress, which has a high predictive value for prognosis (19, 27).

But eventually, it’s inevitable for us to be limited for following reasons. First, this is a retrospective study that focuses on one facet, and we need additive multi-faceted studies to confirm our findings. It is unclear how the included laboratory indicators are related to oxidative stress and its mechanism. Second, the levels of oxidative stress-related biological indicators change over time and were not continuously monitored, and due to clinical limitations, not all laboratory indicators related to oxidative stress were included. Furthermore, We used a variety of ICIs on the patients in this study, which we did not categorize. As far as we know, there are no articles predicting the prognosis of patients receiving immunotherapy for gastric cancer concerning oxidative stress, but this study offers a fresh method on the prognosis of patients with gastric cancer and serves as a resource for the development of oxidative stress and immunotherapy targets.

## Conclusion

GIOSS can predict the clinical outcomes of gastric cancer patients based on oxidative stress levels and simultaneously, demonstrates good predictive performance for gastric cancer patients treated with ICIs, and patients with high GIOSS had poorer clinical outcomes. In a nutshell, the strong predictive power of GIOSS holds promise as a biomarker for measuring the prognosis of cancer patients treated with ICIs.

## Data availability statement

The raw data supporting the conclusions of this article will be made available by the authors, without undue reservation.

## Ethics statement

The studies involving human participants were reviewed and approved by the Ethics Committee of Harbin Medical University Cancer Hospital. Written informed consent for participation was not required for this study in accordance with the national legislation and the institutional requirements.

## Author contributions

Writing-original draft and Writing-review and editing: GD. Data curation and Investigation: HS, RH, RZ, and ZD. Methodology and Supervision: YZ and HP. Resources, Funding acquisition and Project administration: YX and HS.
